# High Performance Liquid Chromatography Determination and Optimization of the Extraction Process for the Total Alkaloids from Traditional Herb *Stephania cepharantha* Hayata

**DOI:** 10.3390/molecules24030388

**Published:** 2019-01-22

**Authors:** Jiao Xiao, Yingni Pan, Lin Zhang, Xia Wang, Yueqing Han, Lu Sun, Gang Chen, Ning Li

**Affiliations:** 1 School of Traditional Chinese Materia Medica, Shenyang Pharmaceutical University, Shenyang 110016, China; xj110121@126.com (J.X.); panyingni@163.com (Y.P.); w1034425846@163.com (L.Z.); hanyueqingsu@163.com (Y.H.); chengang1152001@163.com (G.C.); 2 School of Pharmacy, Shenyang Pharmaceutical University, Shenyang 110016, China; m15702443686@163.com (X.W.); lisunlu@126.com (L.S.)

**Keywords:** *Stephania cepharantha* Hayata, alkaloids, cepharanthine, anti-tumor activity, extraction process

## Abstract

*Stephania cepharantha* Hayata is a traditional Chinese herbal medicine used to treat lung cancer, and its alkaloids, especially cepharanthine (CEP), were reported to be its effective ingredients. Therefore, the extraction of potential antitumor ingredients from the plant was of interest. We first explored the optimized solvent extraction of antitumor agents from *S. cepharantha* Hayata guided by an in vitro antitumor activity assay. The solvent for extraction and its concentration, the liquid to material ratio, extraction duration, particle size, macerating time, and the frequency of extraction were investigated using a single-factor experiment. An orthogonal design (L9, 3^4^) was constructed to determine the suitable extraction conditions. The crude extract was then purified sequentially by macroporous adsorption resins (MR) for the enrichment of CEP. Under these optimal conditions, the yield of total alkaloids in the herbs was 3.4%, whereas the CEP content was 2.9%. Total alkaloids exhibited significant anti-proliferative activities in the A549 cell line. Our study provides means for the further development and use of the antitumor components from *S. cepharantha*, which has potential for application in the pharmaceutical industry.

## 1. Introduction

Lung cancer is the leading cause of cancer mortality in both men and women worldwide [1]. The effectiveness of current treatment is severely limited, with an age-standardized mortality rate of 3 per 10,000 [2]. Chemotherapy is one of the major regimens used to treat most solid tumors. Although the application of cis-diamminedichloroplatinum (cisplatin, CDDP)-based chemotherapy and epithelial growth factor receptor tyrosine kinase inhibitor (EGFR-TKIs) is common, the five-year survival rate of stage III non-small cell lung cancer (NSCLC) patients is about 10%, and that of stage IV NSCLC patients is only 2%. The emergence of drug resistance has been a crucial factor in the failure of clinical treatment [3,4].

Natural phytochemicals obtained from medicinal plants have significantly contributed to the treatment of several human diseases including cancer [5]. Over 3000 plants possessing anticancer properties have been reported [6]. Natural products derived from plants are receiving considerable attention because of their considerable antitumor activities [7]. Investigations showed the critical role of isoquinoline-type alkaloids in cancer therapy [8,9], such as berberine targeted inhibition of prostate cancer [10], tetrandrine with significant anti-proliferation activity against hepatoma cells [11], and berbamine effectively inhibiting leukemia [12], lymphoma [13], myeloma, and lung cancer [14,15]. Cepharanthine (CEP), a bisbenzylisoquinoline alkaloid that was identified from *S. cepharantha* [16], induced apoptosis in human NSCLC [17] and inhibited the growth and metastasis of lung cancer cells [18,19]. CEP exhibited therapeutic effects for lung cancer as a novel autophagy inhibitor [20].

*S. cepharantha* Hayata is a traditional medicinal plant that grows mainly in the south of China. Alkaloids are the major bioactive components in this plant for treatment of parotiditis, gastric ulcers, and leukopenia [21]. However, the application of anti-tumor ingredient CEP has been restricted due to its low abundance in this plant. The conventional methods used to extract effective alkaloids from the *Stephania* genus were developed using extraction with dichloromethane after treatment with hydrochloric acid-water (1:99, *v*/*v*) [22], which was time-consuming (over four hours) and environmentally harmful. Hence, this method was inappropriate for industrial production. Yuan [23] introduced a method for online separation and purification of alkaloids from *S. cepharantha*, applying dynamic pH junction high-speed countercurrent chromatography coupled with microwave-assisted extraction; however, the method had still some difficulties preventing its industrial application. Therefore, it is crucial to find an exhaustive, reproducible, simple, inexpensive, and environmentally friendly approach to extract effective alkaloids from *S. cepharantha*.

In this context, we studied the influence of selected operational variables on the extraction of total alkaloids with antitumor activity from *S. cepharantha*, using the orthogonal test and MR chromatography. The result showed that the maximum extraction rate of total alkaloids was 3.4%, and the content of CEP reached 2.9%. Taken together, the proposed optimized technology is a highly effective method for extraction and purification of total alkaloids from *S. cepharantha*, which market potential for industrial production. The total alkaloids exhibited significant anti-proliferative activities in the A549 cell line.

## 2. Results and Discussion

### 2.1. Optimization of Extraction Process of Crude Extracts

Yield of crude extracts (%) = mass of crude extracts (g)/mass of *S. cepharantha* powder (g) × 100% (1)

The yield of crude extracts was measure via the ratio of crude extracts and raw materials.

#### 2.1.1. Effect of Ethanol Concentration on Extraction Yield and CEP Content from *S. cepharantha*

**Figure 1:**
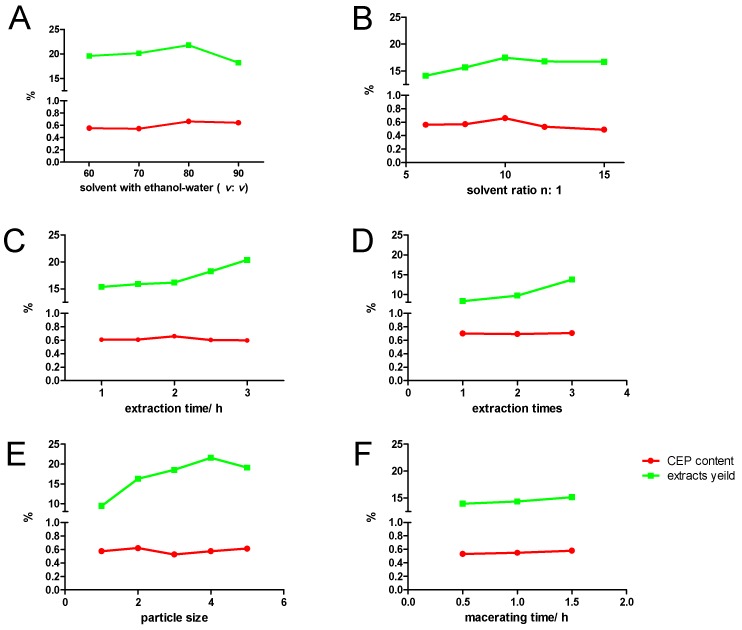
Optimization of the extraction process of crude extractions: (**A**) solvent: 60%, 70%, 80%, and 90% ethanol-water (*v*/*v*); (**B**) liquid to raw material ratio (6:1, 8:1, 10:1, 12:1, and 15:1), (**C**) extraction duration in hours, (**D**) extraction times, (**E**) particle size: 1 for decoction pieces, 2 for coarse powder, 3 for 7 g of powder, 4 for 40 g of the powder, and 5 for 100 g of the powder; and (**F**) macerating time in hours. Green: the weight of extractions (%), Red: the content of CEP in extracts (%).

Usually, selecting appropriate solvents according to the polarity is the first step of the classical process used for extracting natural products from plants. Different concentrations of ethanol-water (60, 70, 80, and 90%, *v*/*v*) were prepared to study the effect of extraction solvent. The ratio of liquid to material was 10:1 (mL/g), heat reflux extraction was sustained for 2 h, and repeated 3 times. Figure 1A shows that the yield of extracts and content of CEP increased with increasing ethanol before the concentration reached 80% However, it declined when the concentration increased from 80 to 90%. Extracting with 80% ethanol-water (*v*/*v*) produced a maximum yield of crude extracts of 21.8%, and the content of CEP was 0.66%. We chose 80% ethanol-water (*v*/*v*) as the primary key for further experiments.

#### 2.1.2. Effect of Ratio of Liquid to Raw Material on Extraction Yield and CEP Content

The screening of the liquid to raw material ratio was an important step [24,25,26]. Crude extracts in raw material cannot be extracted completely with a low ratio of liquid to raw material. Conversely, excessive amounts of solvent would incur higher costs. Therefore, the effect of different ratios (6:1, 8:1, 10:1, 12:1, and 15:1, Figure 1B) on the yield of crude extracts was investigated in this study. We chose 80% ethanol-water (*v*/*v*) as the solvent, then sustained heat reflux extraction for 2 h each time, and repeated 3 times. As seen in Figure 1B, the yield of the crude extracts and the content of CEP reached the critical values of 17.5% and 0.66% at the ratio of 10:1 (g/mL), respectively. Therefore, the ratio of liquid to raw material at 10:1 (g/mL) was selected for further work.

#### 2.1.3. Effect of Extraction Duration on Extraction Yield and CEP Content from *S. cepharantha*

Extraction duration is a parameter that needs to be optimized in order to minimize the energy cost of the process [27]. In the present study, we investigated extraction with 80% ethanol-water (*v*/*v*) for different durations: 1, 1.5, 2, 2.5, and 3 h. As Figure 1C shows, the crude extracts yield increased with increasing extraction time. However, the CEP content increased minimally within 1–1.5 h until the extraction time increased to 1.5–2 h under the same conditions. When the time extended from 2.5 h to 3 h, the content of CEP decreased, which suggests that increased extraction time leads to accelerated dissolution of other ingredients but not alkaloids. Considering production, an extraction duration of 2 h was chosen for subsequent tests.

#### 2.1.4. Effect of the Frequency of Extraction on the Yield of Crude Extracts from *S. cepharantha*

We investigated the effect of another essential factor, the frequency of extraction, on extraction active components from plant materials in this study. The results showed that the yield of crude extracts and the content of CEP increased along with increasing extraction time, indicating that 3 extractions is best (Figure 1D).

#### 2.1.5. Effect of Particle Size on Extraction Yield and CEP Content from *S. cepharantha*

Theoretically, reduction in the particle size could increase extraction effectiveness. Diminishing the size of the particles led to a reduction in the diffusion path, and the larger contact surface area was the reason for the acceleration in the extraction process. However, the smaller particles not only decreased the extraction rate [28] but increased difficulties in the subsequent process of filtering.

Figure 1E shows the effects of different sieved particle sizes on crude extracts yield of *S. cepharantha*, including decoction pieces, coarse powder, and sieving mesh sizes of 7, 40, and 100. The results showed that, compared with decoction pieces, the yield of extracts sharply increased with the particle size in coarse powder, but slowly increased among the groups of coarse powder, sieving mesh size of 7 and 40, and decreased when the sieving mesh size was 100. However, the curves of CEP content fluctuated within the five factors of particle size, and the maximum CEP content obtained with coarse powder particles was 0.57%. According to these results, coarse powder was chosen as a suitable particle size for the preparation of crude extracts and CEP from *S. cepharantha*.

#### 2.1.6. The Effect of Macerating Time on Extraction Yield and CEP Content from *S. cepharantha*

The production of the extraction involves soaking of the tubers [29]. The results, shown in Figure 1F, indicated that the maximum extraction rate was 15.2% and the CEP content was 0.58%, which was obtained by soaking for 1.5 h.

From the results mentioned above, we employed extract yield and CEP content as an index, and selected four factors—The ratio of liquid to raw material B (8:1, 10:1, and 12:1), macerating time F (0, 1, and 1.5 h), the frequency of extraction D (2, 3, and 4 times), and extraction duration C (1, 2, and 3 h)—to adopt an orthogonal L9 (3^4^) test design. The orthogonal experimental design and results are shown in Table 1 based on three experimental repetitions performed in parallel. Intuitive analysis showed that the effect of various factors on the yield followed the order D > C > F > B. The frequency of extraction played the most important role on extraction efficiency, followed by extraction duration, macerating time, and then liquid to material ratio. The effect of various factors on the content of CEP followed the order B > F > C > D. Along with the single-factor tests, we determined that the best conditions were B_3_F_3_D_3_C_3_.

The problems with the orthogonal method were the time consumption and large amounts of volatile organic solvents, which are not suitable for industrial production. Therefore, in order to propose a better scheme for establishment more scientific quality standards, we compared three projects to find the best extraction conditions.

Scheme 1 was B3F3D3C3.

For Scheme 2, a certain amount of *S. cepharantha* powder was macerated with a 12-fold amount of 80% ethanol-water (*v*/*v*) for 1.5 h, and extracted with heat reflux for 3 h. We filtered the solution, and the residues of herbs were extracted with a 10-fold amount of 80% ethanol-water (*v*/*v*) for 2 h, then we repeated the process once.

For Scheme 3, we followed the same process for Scheme 2, but the extraction process was repeated 3 times.

Comparing Schemes 2 and 3, we observed no significant effect on the experimental results with the increase in extraction repetitions. The results were almost the same for Schemes 1 and 2 except for the time required. Hence, the conditions in Scheme 2 are more suitable, as a simple, time-saving, simple, and inexpensive process. The optimal conditions for obtaining the crude extracts from *S. cepharantha* were as follows: powdered *S. cepharantha* (5 g) was macerated with 60 mL of ethanol-water (80:20, *v*/*v*) for 1.5 h, then extracted under heat reflux for 2 h, and the supernatant fraction (I) was filtered. The residue recovered was refluxed with 50 mL of ethanol-water (80:20, *v*/*v*) solution for 2 h (II), and then repeated once (III). The results showed that the yield of crude extracts from *S. cepharantha* was 18.6%, whereas the CEP was about 0.53%, which agreed with the repeated experiments.

### 2.2. Optimization of Purification Process of Total Alkaloids

MR has been widely used in the separation and enrichment of bioactive compounds from many natural products for simple, highly efficient, inexpensive, and environmentally friendly [30,31,32,33,34,35,36].

Our target compound’s (CEP) inability to dissolve in pure water or in low concentrations of ethanol-water solution caused difficulties when determining the adsorption data for CEP on resins when using the normal method. Hence, the crude extracts samples were dissolved with ethanol and adsorbed with MR. After drying the mixture at room temperature, we eluted it separately with different concentrations of ethanol-water solvent.

Volume (mL) = 2.5 × (the weight of MR + the weight of sample) mL(2)

Yield of total alkaloids (%) = mass of total alkaloids (g)/mass of crude extracts (g) × 100%(3)

The yield of total alkaloids was obtained by the value of total alkaloids over crude extracts.

#### 2.2.1. Effect of the Different Types of MR on Total Alkaloids Yield and CEP Content from *S. cepharantha*

We investigated the ability of three types of MR, D101, HPD-100, and AB-8, to obtain CEP and total alkaloid from crude extracts. A certain amount of crude extracts was adsorbed with a 4-fold amount of MR and then eluted via 30:70 (*v*/*v*), 70:30 (*v*/*v*), and 95:5 (*v*/*v*) for 10 volumes. There was no significant distinction for the three different resins; therefore, we selected D101 resin as the most appropriate resin for the separation of CEP.

#### 2.2.2. Effect of the Elution Solvent on Total Alkaloids Yield and CEP Content from *S. cepharantha*

As for alkaloids, the pH value of the elution solvent was a critical factor and considerably influenced ion behavior, extraction selectivity, and yield of target compounds. To determine optimal operation conditions, a series of solutions with different pH values were used to purify CEP, as shown in Table 2. After elution via ethanol-water-triethylamine (30:65:5, *v*/*v*/*v*), ethanol-water-formic acid (70:25:5, *v*/*v*/*v*), and ethanol-water (95:5, *v*/*v*), we found that the CEP content reached a maximum in 70% elution, which was the total alkaloids parts. Therefore, the elution solvent mentioned above was applied in the subsequent work.

#### 2.2.3. Effect of Volumes on Total Alkaloids Yield and CEP Content from *S. cepharantha*

We also investigated the effect of different volumes of solvent (8, 10, and 12 mL) on total alkaloids yield and CEP content. The result (Table 3) showed that the CEP content increased with increasing volume. However, the yield of total alkaloids increased from 8 to 10 and then decreased with further volume increases. Hence, we selected 10 as the suitable volume.

#### 2.2.4. Effect of Sample MR Ratios on Total Alkaloids Yield and CEP Content from *S. cepharantha*

We studied different sample–MR ratios including 3, 4, and 5 (Table 4). As a result, the content of target compounds and the yield of total alkaloids yield peaked when the sample–MR ratio was 1:4. Therefore, we chose the weight of MR as 4-fold higher than the sample for further study.

Determined from the results, the D101 type of MR produced optimum absorption and elution parameters. The optimum elution process of the total alkaloids from *S. cepharantha* was as follows: we used a certain amount of crude extract with quadruple the amount of MR, and eluted using ethanol-water-triethylamine (30:65:5, *v*/*v*/*v*), ethanol-water-formic acid (70:25:5, *v*/*v*/*v*), ethanol-water (95:5, *v*/*v*), and flushing 10 column volumes. The results showed that the yield of total alkaloids was 3.4%, whereas the CEP was about 2.9%, according to the repeated experiments.

### 2.3. HPLC analysis of CEP in Crude Extracts and Total Alkaloids

**Figure 2:**
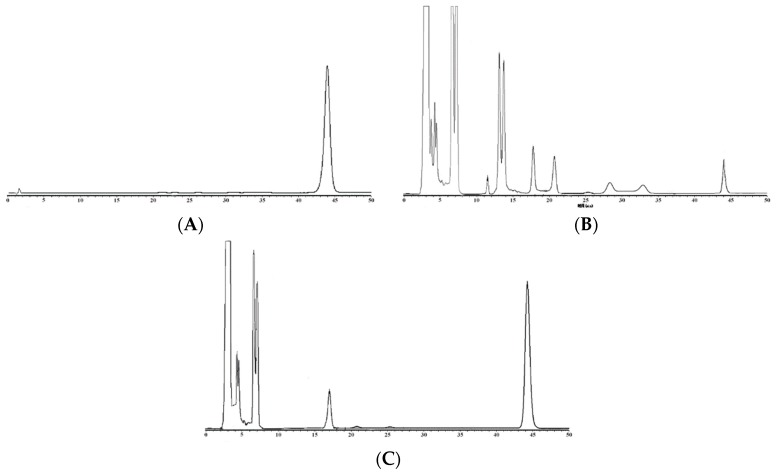
HPLC chromatograms of CEP (**A**) crude extracts (**B**) and total alkaloids (**C**) from *S. cepharantha* Hayata marked in star-shaped.

The CEP content (Figure 2A) in crude extracts (Figure 2B) and total alkaloids (Figure 2C) from *S. cepharantha* were analyzed by HPLC, as shown in Figure 2. The CEP content in total alkaloids (2.9%) remarkably increased compared to crude extracts (0.1%).

### 2.4. Assay Cytotoxic in A549 Cells

**Figure 3:**
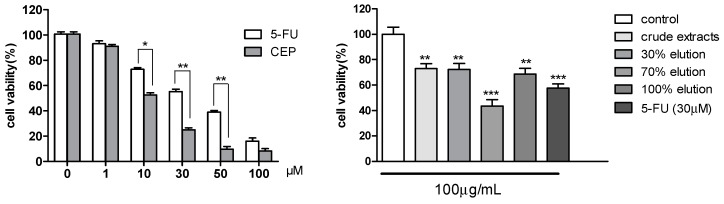
Cytotoxicity assay of CEP, crude extracts, and elutions by MTT in A549 cells. Results are the mean ± SD from three independent experiments. Significance: * *p* < 0.05, ** *p* < 0.01, and *** *p* < 0.001 compared to control groups.

Crude extracts and all fractions were evaluated for their anti-proliferative activity. The 70% elution, the most effective fraction, exhibited significant results at 100 μg/mL in A549 cells (Figure 3). We found that CEP showed dose-dependent anti-proliferative activity, which was consistent with the result that the CEP content was highest in the 70% elution.

## 3. Material and Methods

### 3.1. Chemicals and Reagents

*S. cepharantha* was collected from Yunnan province in China, identified by Professor Yingni Pan (Shenyang Pharmaceutical University, Shenyang, China). The reference standard of CEP (purity > 98%) was purchased from Tianjin Yifang Science and Technology Co., LTD (Tianjin, China), and confirmed by nuclear magnetic resonance (NMR) analysis. Methanol and acetonitrile were high performance liquid chromatography (HPLC) grade (Fisher, Fair Lawn, NJ, USA). Purified water was purchased from Watsons Co. Ltd. (Guangdong, China). For sample extraction, water, ethanol, and methanol were both of analytical grade.

Three types of MR, AB-8, HPD-100, and D101, were purchased from Cangzhou Bon Adsorber Technology Co., LTD (Hebei, China). The physical properties of those resins are summarized in the Table 5. All adsorbents required pretreatment by 95% ethanol-water (*v*/*v*) to remove monomers and porogenic agents trapped inside the pores before use, and then dried at room temperature. 

### 3.2. Conditions for Determining the Content of CEP by HPLC

We used a Shimadzu series LC-10AT HPLC (Shimadzu Corporation, Shanghai, China), using a Shim-pack PREP-ODS C18 column (250 mm × 4.6 mm, 5 μm, Shimadzu, Shanghai, China), with the temperature maintained at 30 °C. After investigating various mobile phases in different combinations, we selected an isocratic method, with the mobile phase consisting of 52% acetonitrile-water, containing 0.5% triethylamine, and 0.01% orthophosphoric acid (*v*/*v*), at a flow rate of 1 mL/min. The ultraviolet (UV) detection (Shimadzu, Shanghai, China) was achieved at 282 nm. The mobile phases were filtered by using a 0.45 μm filter and degassed by sonication for 10 min before use.

### 3.3. Preparation of Reference Standards

The standard stock solution of CEP was prepared by dissolving it in methanol to produce a solution with a concentration of 1.00 mg/mL. The working solution was prepared by diluting the stock solution with acetonitrile-water (52:48, *v*/*v*).

### 3.4. Preparation of Samples Solution

All the samples, such as crude extracts and total alkaloids, were dissolved in acetonitrile-water (52:48, *v*/*v*), then filtered through a 0.22-μm filter prior, taking 10 µL of filtrate injected into the HPLC for the determination of CEP content.

### 3.5. Method Validation and Quantitative Analysis

The proposed method was validated in terms of linearity, limits of detection and quantification (LOD and LOQ, respectively), precision (intra- and inter-day variations), repeatability, stability, and recovery. Intra-day precision was examined using six replications of standard solutions within one day, whereas inter-day precision was determined by performing evaluations on three consecutive days. The stability test was performed by analyzing the sample at 0, 2, 4, 8, 24, and 48 h, and calculating the relative standard deviations (RSDs) for the peak area ratio of each sample. In order to confirm the repeatability of the developed method, six samples of solutions were prepared independently from the same CEP sample.

The peak areas of standards were used to generate a linear regression curve, which presented good linear correlations (*R*^2^ = 0.9997) within the tested concentrations. The linear range of this method was 9–216 µg/mL. Intra- and inter-day RSDs were < 2.36% and 3.17%, respectively. The six independently prepared samples demonstrated good repeatability (RSD = 1.65%). Finally, the stability of the sample solution was tested at room temperature (RSD < 1.74%). The mean recoveries of CEP from the test samples were found to be in the range of 96.21 to 103. Of the samples, 87% had RSD values less than 4.72%, suggesting the good recovery ability of this developed method.

### 3.6. Assay Cytotoxic in A549 Cells

The A549 cells were seeded in a 96-well plate at a density of 2.5 × 10^4^/mL and cultured for 48 h to form a monolayer. Samples were added into the plate in the following concentrations: 100, 30, 50, 10, and 1 g/mL, and CEP and 5-FU were 100, 30, 50, 10, and 1 μM. After cultured for 24 h, 50 μL MTT (2 mg/mL) was added in the next 4 h. The supernatant was then removed and 100 μL dimethyl sulfoxide (DMSO) was added. The absorbance at 490 nm (OD_490_ value) was measured by an ELISA microplate reader (Power Wave XS2, Bio Tek, Winooski, VT, USA) after shaking 5 min (Orbital Shaker TS-1, Decolorizing Orbital Shaker, Kylin-Bell Lab Instruments Co., Ltd., Jiang Su, China). The OD_490_ value was in accordance with the number of live cells: the larger the OD_490_ value was, the more numerous the live cells.

## 4. Conclusions

CEP, a bisbenzylisoquinoline type of alkaloid that was first reported in the *Stephania* genus, exhibited a significant therapeutic effect for lung cancer. In this work, HPLC chromatography determination and optimization of the solvent extraction of CEP from *S. cepharantha* Hayata was studied. Based on the results of a single-factor experiment, we constructed an orthogonal design (L9, 3^4^) to determine the suitable extraction conditions. The crude extract was then purified sequentially by MR for the enrichment of CEP. The yield of total alkaloids was 3.4%, whereas the CEP was about 2.9%. The fraction of total alkaloids, which contained the CEP, displayed remarkable anti-proliferative activity in A549 cells. This suggested a potential method to enrich the active components from *S. cepharantha* Hayata for large-scale industrial production.

## Figures and Tables

**Table 1:** Design and results of orthogonal test of extraction process optimization on extraction yield and CEP content from *Stephania cepharantha*.

No.	B	F	D	C	Weight (%)	CEP Content (%)
1	1:8	0.5	2	1	19.06	0.4918
2	1:8	1	3	2	17.83	0.4919
3	1:8	1.5	4	3	24.94	0.5005
4	1:10	0.5	3	3	19.67	0.5351
5	1:10	1	4	1	19.23	0.5651
6	1:10	1.5	2	2	18.07	0.5110
7	1:12	0.5	4	2	21.36	0.5497
8	1:2	1	2	3	19.67	0.5693
9	1:12	1.5	3	1	18.67	0.5751
K_1_	0.4947	0.5255	0.5240	0.5440	Range of CEP	
K_2_	0.5370	0.5421	0.5340	0.5175		
K_3_	0.5646	0.5288	0.5384	0.5349		
R	0.0699	0.0165	0.0100	0.0265		
K_1_	20.61	20.03	18.93	18.99	Range of weight	
K_2_	18.99	18.91	18.73	19.09		
K_3_	19.90	20.56	21.84	21.43		
R	1.62	1.65	3.12	2.44		

**Table 2:** Effect of the elution solvent on total alkaloids yield and CEP content yield from *S. cepharantha*.

No.	Elution	Yield (%)	Content (%)
1	ethanol:water (30:70, *v*/*v*)	9.65	0.1390
	ethanol:water (70:30, *v*/*v*)	2.02	0.3160
	ethanol:water (95:5, *v*/*v*)	1.50	2.5837
2	ethanol:water:triethylamine (30:65:5, *v*/*v*/*v*)	13.19	0.0513
	ethanol:water:triethylamine (70:25:5, *v*/*v*/*v*)	2.43	0.1623
	ethanol:water (95:5, *v*/*v*)	1.92	2.5183
3	ethanol:water:triethylamine (30:65:5, *v*/*v*/*v*)	12.19	0.0848
	ethanol:water:formic acid (70:25:5, *v*/*v*/*v*)	3.88	2.7246
	ethanol:water (95:5, *v*/*v*)	0.86	0.1137

**Table 3:** Effect of the volume on total alkaloids yield and CEP content from *S. cepharantha*.

Volume (mL)	Elution	Yield (%)	Content (%)
8	ethanol:water:triethylamine (70:25:5, *v*/*v*/*v*)	2.69	2.3519
10	ethanol:water:triethylamine (70:25:5, *v*/*v*/*v*)	3.98	2.7528
12	ethanol:water:formic acid (70:25:5, *v*/*v*/*v*)	2.91	2.9474

**Table 4:** Effect of the solid–liquid ratio on total alkaloids yield and CEP content from *S. cepharantha*.

Ratio	Elution	Yield (%)	Content (%)
3	ethanol:water:triethylamine (70:25:5, *v*/*v*/*v*)	3.65	2.5005
4	ethanol:water:triethylamine (70:25:5, *v*/*v*/*v*)	3.42	2.9503
5	ethanol:water:formic acid (70:25:5, *v*/*v*/*v*)	2.06	2.3959

**Table 5:** Result of adsorption and desorption capacities of different macroporous resins.

Name	Polarity	Particle Diameter (mm)	Surface Area (m^2^/g)	Pore Diameter (nm)
HPD100	Nonpolar	0.3–1.20	650–700	8.5–9.0
AB-8	Weak polar	0.3–1.25	480–520	13.0–14.0
D101	Nonpolar	0.3–1.25	≥400	10.0–11.0
